# Health utilities in Chinese patients with coronary heart disease and impaired glucose tolerance (ACE): A longitudinal analysis of a randomized, double‐blind, placebo‐controlled trial

**DOI:** 10.1111/1753-0407.13294

**Published:** 2022-07-25

**Authors:** José Leal, Frauke Becker, Lee‐Ling Lim, Rury R. Holman, Alastair M. Gray

**Affiliations:** ^1^ Health Economics Research Centre University of Oxford Oxford UK; ^2^ Department of Medicine University of Malaya Kuala Lumpur Malaysia; ^3^ Diabetes Trials Unit University of Oxford Oxford UK

**Keywords:** cerebrovascular diseases, economics, prediabetic state, quality of life, vascular diseases, 血管疾病, 经济学, 糖尿病前期状态, 生活质量, 脑血管疾病

## Abstract

**Background:**

We estimate health‐related quality of life and the impact of four cardiovascular events (myocardial infarction [MI], stroke, congestive heart failure, angina) and gastrointestinal events in 6522 Chinese patients with coronary heart disease (CHD) and impaired glucose tolerance (IGT) participating in the Acarbose Cardiovascular Evaluation (ACE) trial.

**Methods:**

Health‐related quality of life was captured using the EuroQol‐5 Dimension‐3 Level (EQ‐5D‐3L), with data collected at baseline and throughout the trial. Multilevel mixed‐effects linear regression with random effects estimated health‐related quality of life over time, capturing variation between hospital sites and individuals, and a fixed‐effects linear model estimated the impact of cardiovascular and gastrointestinal events.

**Results:**

Patients were followed for a median of 5 years (interquartile range 3.4‐6.0). The average baseline EQ‐5D score of 0.930 (SD 0.104) remained relatively unchanged over the trial period with no evidence of statistically significant differences in EQ‐5D score between randomized treatment groups. The largest decrement in the year of an event was estimated for stroke (−0.107, *P* < .001), followed by heart failure (−0.039, *P* = .022), MI (−0.021, *P* = .047), angina (−0.012, *P* = .047), and gastrointestinal events (−0.005, *P* = .430). MI and stroke reduced health‐related quality of life beyond the year in which the event occurred (−0.031, *P* = .006, and −0.067, *P* < .001, respectively).

**Conclusions:**

Acarbose treatment had no impact on health‐related quality of life in ACE trial participants with CHD and IGT. Events such as MI, stroke, heart failure, and angina reduce health‐related quality of life around the time they occurred, but only MI and stroke impacted on longer‐term health‐related quality of life.

## Graphical abstract

**Highlights**

- The Acarbose Cardiovascular Evaluation (ACE) trial assessed the effects of acarbose in patients with coronary heart disease (CHD) and impaired glucose tolerance (IGT) in China.
- Acarbose treatment did not impact on health‐related quality of life in ACE trial participants with CHD and IGT.
- Myocardial infarction (MI), stroke, heart failure, and angina were found to reduce health‐related quality of life around the time they occurred; only MI and stroke may impact on longer‐term health‐related quality of life.

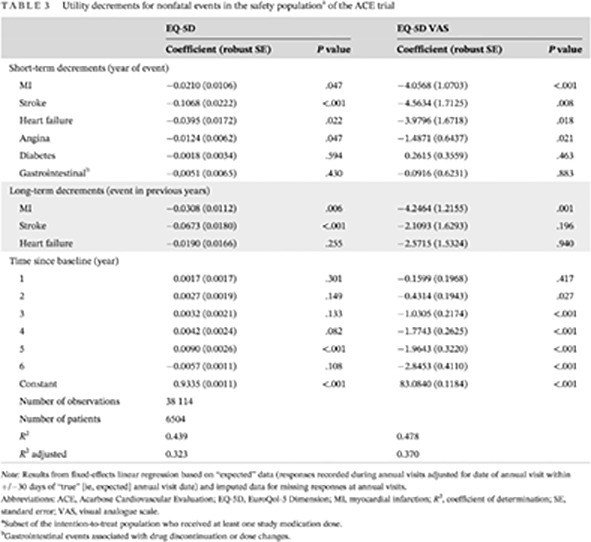

## 摘要

**背景:**

我们评估了参加阿卡波糖心血管评估(ACE)试验的6,522名中国冠心病(CHD)和糖耐量异常(IGT)患者的健康相关生活质量，以及四种心血管事件(心肌梗死(MI)、中风、充血性心力衰竭、心绞痛)和胃肠道事件的影响。

**方法:**

使用EuroQol‐5 Dimension‐3 Level(EQ‐5D‐3L)获取与健康相关的生活质量信息，收集基线和整个试验过程中的数据。运用具有随机效应的多水平混合效应线性回归估计随时间进展的与健康相关的生活质量，并记录医院和个人之间的差异，使用固定效应线性模型估计心血管和胃肠道事件的影响。

**结果:**

患者平均随访5年(四分位差3.4~6.0)。在试验期间，平均基线EQ‐5D得分为0.930(SD 0.104)保持相对不变，没有证据表明随机治疗组之间的EQ‐5D得分在统计学上有显著差异。在事件发生的一年中，使健康相关的生活质量下降幅度最大的是中风(‐0.107，p<0.001)，其次是心力衰竭(‐0.039，p=0.022)、心肌梗塞(‐0.021，p=0.047)、心绞痛(‐0.012，p=0.047)和胃肠道事件(‐0.005，p=0.430)。心肌梗死和中风降低了事件发生后一年与健康相关的生活质量(分别为0.031，p=0.006和‐0.067，p<0.001)。

**结论:**

阿卡波糖治疗对患有冠心病和糖耐量异常的ACE试验参与者的健康相关生活质量没有影响。心肌梗死、中风、心力衰竭和心绞痛等事件在发生时会降低与健康相关的生活质量，但只有心肌梗死和中风会对长期的健康相关生活质量产生影响。

## 1 INTRODUCTION

The global prevalence of diabetes in adults aged between 20 and 79 years is projected to rise from 463 million in 2019 to 700 million by 2045.^1^ Elevated glycemic levels have been found to increase the risk of microvascular and macrovascular complications,^2^ and hyperglycemia is considered the main contributor to the onset of prediabetes.^3^, ^4^ Evidence from randomized controlled trials suggests that lifestyle interventions and oral glucose‐lowering drugs can effectively delay or prevent progression from prediabetes to diabetes.^5^, ^6^, ^7^, ^8^, ^9^ However, policy decisions considering the cost‐effectiveness of preventative interventions in populations with prediabetes require the assessment of costs and outcomes (including health‐related quality of life) over a lifetime period.

While individual sociodemographic characteristics (eg, age, gender) affect health‐related quality of life in general, cardiovascular events such as myocardial infarction (MI), stroke, congestive heart failure (CHF), as well as angina have been shown to affect individual health‐related quality of life significantly.^10^, ^11^, ^12^, ^13^, ^14^ Associations between events and health‐related quality of life have typically been estimated using cross‐sectional data.^13^ However, such analyses will attribute differences in health‐related quality of life between patients who do and do not experience an event at any one time to the impact of the event itself. Therefore, cross‐sectional analyses could lead to biased estimates as potentially unobserved heterogeneity between patients cannot be controlled for. Studies using longitudinal data^15^, ^16^ have overcome this limitation, controlling for characteristics that may affect individual health‐related quality of life before patients experience an event, thereby isolating the event‐related impact in health‐related quality of life from other patient‐specific effects.

The Acarbose Cardiovascular Evaluation (ACE) trial, was a multicenter, randomized, double‐blind, placebo‐controlled trial that assessed the effects of acarbose, an α‐glucosidase inhibitor, in patients with coronary heart disease (CHD) and impaired glucose tolerance (IGT) in China.^7^ The primary objective of the ACE trial was to determine whether adding acarbose to optimized cardiovascular therapy could reduce cardiovascular‐related morbidity and mortality compared to placebo in a Chinese healthcare setting. The trial showed that acarbose did not reduce the risk of major adverse cardiovascular events in Chinese patients with CHD and IGT, but did reduce the incidence of diabetes.

We used data from the ACE trial^7^ to estimate health‐related quality of life in Chinese patients with CHD and IGT over time, controlling for individual sociodemographic characteristics and treatment assignment. Furthermore, we estimated the impact of four cardiovascular events (MI, stroke, CHF, angina) and gastrointestinal adverse events on health‐related quality of life over a maximum of a 6‐year period.

## 2 METHODS

### 2.1 Study population

The ACE intention‐to‐treat population included 6522 participants with CHD and IGT recruited from 176 hospital outpatient clinics in China who were randomly assigned to oral acarbose 50 mg three times a day (n = 3272) or matched placebo (n = 3250). Median follow‐up was 5.0 years (interquartile range 3.4‐6.0) in both trial arms, providing repeated within‐trial health‐related quality of life measures per participant. The full description of the trial, including patient flow diagram, baseline characteristics, and results have been published previously.^7^ At baseline, 43% of patients had prior MI, 7% stroke, 4% CHF, and 63% either stable or unstable angina. The average EuroQol‐5 Dimension (EQ‐5D) score in both arms was 0.930 (SD 0.104) at baseline. The ACE study protocol was approved by the University of Oxford Tropical Research Ethics Committee and by central or local ethics committees (as appropriate) at participating sites.

### 2.2 Health‐related quality of life

Health‐related quality of life was captured using the EuroQol‐5 Dimension‐3 Level (EQ‐5D‐3L) questionnaire.^17^ This was administered to participants at baseline and each annual visit and occasionally during 4‐monthly visits. Responses to the five dimensions of the EQ‐5D‐3L questionnaire (mobility, self‐care, usual activities, pain/discomfort, and anxiety/depression) were converted into health utilities using a Chinese value set.^18^ Data used for the longitudinal analyses were truncated at the end of year 6 of follow‐up due to the limited number of observations for years 7 to 8. Partially completed EQ‐5D‐3L questionnaires were considered missing.

The pattern and quantity of missing data for health utilities were assessed^19^ to determine the appropriate method for imputing missing data. Conditional on the patterns of missing data being suggestive of missing at random, we aimed to impute EQ‐5D‐3L utility scores and visual analogue scale (VAS) scores at annual visits up to year 6 of follow‐up using multiple imputation^20^ with predictive mean matching of 10 nearest neighbors.^21^ The imputation model included sex, site, region, events (conditional on being at risk at each year of follow‐up), as well as age, risk factors (eg, glycosylated hemoglobin, low‐density lipoprotein, smoking status, etc), and comorbidities at baseline. Missing observations included data for those patients who had withdrawn from the study but who were contacted for a trial close‐out visit.

### 2.3 Statistical analyses

#### 2.3.1 Health utilities in the acarbose group compared with the placebo group

Using intention‐to‐treat analysis, we compared EQ‐5D‐3L utility scores between trial arms using a multilevel mixed‐effects linear regression with random effects at individual and site level.^22^, ^23^, ^24^ We assumed that patients were clustered within site level and captured between‐patient and between‐site variation as random intercepts. Fixed effects included treatment assignment, time since baseline (in years), an interaction term between treatment assignment and time since baseline, baseline EQ‐5D‐3L utility score, and participants' baseline characteristics (age, sex).

The base case analysis of health utility data used all observed responses recorded during routine and annual visits with no imputation of missing data (model 1). For annual visit data, we assumed that each record date matched with the expected date of the visit. In order to account for variation in annual visit dates across patients as well as missing data, sensitivity analyses were run using the mixed‐effects model with different data specifications:

- Available case analysis on expected data: using observed data that matched the expected date of annual visits (within +/−30 days around the expected annual visit date). If the recorded annual visit date was outside this range, we used linear interpolation between available data points from the patient's routine and other annual visits to calculate an EQ‐5D utility score corresponding with the expected date of the annual visit. Mean imputation was used for missing data on baseline EQ‐5D to ensure that baseline values remained independent of treatment allocation and post‐baseline outcomes (model 2).
- Imputation of missing data: multiple imputation of missing annual EQ‐5D utilities using predictive mean matching with 10 nearest neighbors; mean imputation of missing baseline utilities (model 3). Estimates derived from each imputed dataset were combined using Rubin's Rule.^25^
- Complete patient analysis on observed data: subset of patients with complete EQ‐5D data from all annual visits during their follow‐up period (no imputation of missing values) (model 4).

#### 2.3.2 Impact of diabetes and cardiovascular and gastrointestinal adverse events on health utility

Following multiple imputation, we estimated the impact of diabetes diagnosis and nonfatal cardiovascular and gastrointestinal events on health‐related quality of life using a fixed‐effects linear model.^26^ This type of model allows for robust estimation of health‐related quality of life decrements in presence of unobserved patient heterogeneity. The fixed‐effects model captured within‐patient effects, estimating the associations between EQ‐5D utility score, diabetes diagnosis, and cardiovascular (MI, stroke, CHF, angina) and gastrointestinal adverse events while controlling for individual time‐invariant characteristics (eg, sex, ethnicity). Data on gastrointestinal adverse events associated with drug discontinuation or dose changes were collected in the safety population of the ACE trial (n = 6504), a subset of the ACE trial population who received at least one study medication dose, and we used this population in our analysis. Time‐fixed effects were included using binary covariates for each wave of data (in annual intervals), and they allowed capturing the average trend affecting all patients over time. Previous studies found evidence for differences between short‐term and long‐term health‐related quality of life decrements following cardiovascular complications,^15^ suggesting that the initial decrement associated with an acute event might decrease over time. Hence, we estimated short‐term decrements by including events occurring in the same year as the EQ‐5D measurement and estimated long‐term decrements by means of the event history, that is, events having occurred within‐trial in the years prior to the EQ‐5D measurement. As sensitivity analysis we used the EQ‐5D VAS score instead of the EQ‐5D utility score as the dependent variable and ran the model described above. Estimates derived from each imputed dataset were combined using Rubin's Rule. Statistical significance levels, coefficient of determination (*R* ^2^) and adjusted *R* ^2^ as well as Akaike and Bayesian information criteria (AIC, BIC)^27^, ^28^ across imputed datasets were used to determine the best‐fit model. A Hausman test^29^ was used to test the fixed‐effects model against an alternative random‐effects model.

All analyses were run in Stata version 17.0, using the *mi impute pmm* (multiple imputation using predictive mean matching), *mixed* (multilevel mixed‐effects linear regression), and *xtreg* (fixed‐effects linear model) commands.

## 3 RESULTS

### 3.1 Sample

**FIGURE 1:**
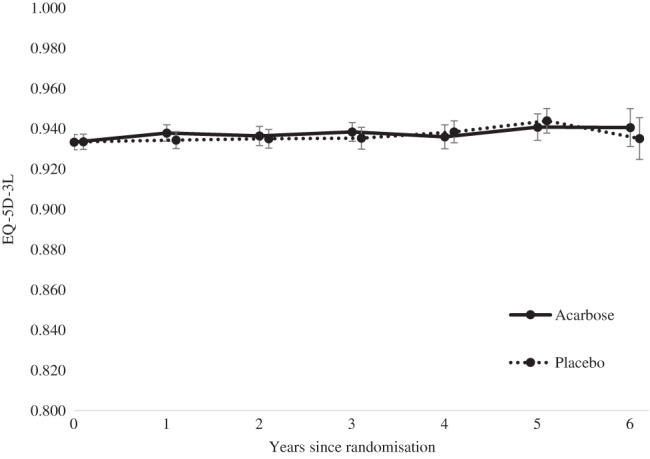
EQ‐5D‐3L scores (mean, 95% CI) from baseline to year 6 of trial follow‐up based on observed data (ie, responses recorded during routine and annual visits, no imputation of missing data)

The majority of participants reported “no problems” for all dimensions of the EQ‐5D (mobility, self‐care, usual activities, pain/discomfort, anxiety/depression) at each time point (see Tables S1‐S5). The average baseline EQ‐5D utility score of 0.930 (SD 0.104) remained relatively unchanged over the trial period. This trend was the same across both trial arms (see Figure 1; Figures S1, S2 and Tables S6‐S8). The EQ‐5D VAS suggested a small decrease in health‐related quality of life in both trial arms from baseline (see Table S9; Figure S3).

The amount of missing data ranged between 6% and 44% for EQ‐5D data observed during years 1 to 6 of the follow‐up period (see Tables S1‐S5). Most missing data were due to participants not attending annual follow‐up visits after discontinuation, for whom, however, data were available from a final trial close‐out visit. The patterns of missing data were suggestive of missing at random with lagged values and events being significantly associated with the probability of data missing (via logistic models). Therefore, missing data on health utilities at annual visits were imputed using multiple imputation.

### 3.2 Health utilities in the acarbose group compared with the placebo group

Table 1 shows that there was no evidence of a statistically significant difference in EQ‐5D utility scores between trial arms in any of the four data specifications: that is, (1) available case (observed data), (2) available case (expected data), (3) multiple imputation, and (4) complete patient case. The differences in utility scores remained statistically insignificant when treatment allocation was not interacted with time since randomization. Following multiple imputation (model 3), the mean difference in EQ‐5D utility was 0.002 (95% CI, −0.002 to 0.007; *P* = .278) between the acarbose and placebo groups. Baseline EQ‐5D had a significant positive association with EQ‐5D over time (*P* < .001), while baseline age (*P* < .001) and women (*P* < .001) were found to be negatively correlated with EQ‐5D.

**TABLE 1:** Results from multilevel mixed‐effects linear regression with random effects on individual and site (years 0‐6)

	Model 1		Model 2		Model 3		Model 4	
	Available case (observed data)^a^		Available case (expected data)^b^		Multiple imputation^c^		Complete patient^d^	
Number of observations	25 963		26 138		38 144		14 558	
Number of patients	6409		6522		6522		2792	
*Fixed effects*	*Coefficient (robust SE)*	*P value*	*Coefficient (robust SE)*	*P value*	*Coefficient (robust SE)*	*P value*	*Coefficient (robust SE)*	*P value*
Acarbose	0.0016 (0.0013)	.221	0.0013 (0.0013)	.293	0.0024 (0.0023)	.278	0.0006 (0.0021)	.782
Baseline EQ‐5D	**0.5138 (0.0153)**	<.001	**0.5156 (0.0154)**	<.001	**0.3919 (0.0138)**	<.001	**0.4620 (0.0223)**	<.001
Baseline age	**−0.0013 (0.0001)**	<.001	**−0.0012 (0.0001)**	<.001	**−0.0012 (0.0001)**	<.001	**−0.0013 (0.0002)**	<.001
Female	**−0.0099 (0.0021)**	<.001	**−0.0096 (0.0020)**	<.001	**−0.0106 (0.0019)**	<.001	**−0.0102 (0.0031)**	<.01
Years since baseline	0.0007 (0.0009)	.423	0.0007 (0.0009)	.423	0.0001 (0.0009)	.920	0.0003 (0.0010)	.855
Years since baseline × acarbose	−0.0008 (0.0007)	.272	−0.0006 (0.0007)	.373	−0.0002 (0.0010)	.865	0.0001 (0.0010)	.947
*Random effects*	*Estimate (robust SE)*	*95% CI*	*Estimate (robust SE)*	*95% CI*	*Estimate (robust SE)*	*95% CI*	*Estimate (robust SE)*	*95% CI*
Site	0.0002 (0.0000)	0.0002‐0.0003	0.0002 (0.0000)	0.0002‐0.0003	0.0180 (0.0017)	0.0150‐0.0216	0.0003 (0.0001)	0.0002‐0.0004
Patient	0.0014 (0.0001)	0.0012‐0.0016	0.0014 (0.0001)	0.0012‐0.0016	0.0377 (0.0015)	0.0348‐0.0409	0.0016 (0.0002)	0.0013‐0.0019

*Note:* Statistically significant (5%) values in bold.

Abbreviation: EQ‐5D, EuroQol‐5 Dimension.

^a^ Observed data from responses recorded during annual and routine visits.

^b^ Expected data (responses recorded during annual visits adjusted for date of annual visit within +/−30 days of “true” annual visit date) and observed data as recorded during routine visits.

^c^ Expected data (responses recorded during annual visits adjusted for date of annual visit within +/−30 days of “true” annual visit date and observed data as recorded during routine visits) and imputed data for missing responses at annual visits.

^d^ Observed data on a subset of patients for whom EQ‐5D data were available from each annual visit during their follow‐up period.

### 3.3 Utility values for cardiovascular and gastrointestinal adverse events

The number of nonfatal cardiovascular events (MI, stroke, heart failure, angina), gastrointestinal adverse events associated with drug discontinuation or dose changes, and the average number of years available to capture cardiovascular event history during the first 6 years of the trial follow‐up period are reported in Table 2. In the safety population (n = 6504) and during the first 6 years of the trial follow‐up, the most frequently recorded events were new‐onset diabetes (n = 943), followed by (stable or unstable) angina (n = 385), gastrointestinal events associated with drug discontinuation or dose changes (n = 363), MI (n = 206), CHF (n = 131), and stroke (n = 124). Most MI and angina events observed during the trial follow‐up occurred in patients with a baseline history of those events (MI 58%, angina 65%), while most strokes (85%) and heart failure episodes (80%) were recorded as first events.

**TABLE 2:** Number of nonfatal events (years 0‐6 of follow‐up) and corresponding duration of history in the safety population^a^ of the ACE trial

Nonfatal events	Total	Without baseline history of event (%)	With baseline history of event (%)	Average number of years with history of event during trial follow‐up (SD)
MI	206	86 (42%)	120 (58%)	2.1 (1.7)
Stroke	124	106 (85%)	18 (15%)	2.0 (1.9)
Heart failure	131	105 (80%)	26 (20%)	2.0 (1.8)
Angina	385	134 (35%)	251 (65%)	2.6 (1.8)
Diabetes	943			2.9 (1.6)
Gastrointestinal^b^	363			
Number of patients	6504			

Abbreviations: ACE, Acarbose Cardiovascular Evaluation; MI, myocardial infarction; SD, standard deviation.

^a^ Subset of the intention‐to‐treat population who received at least one study medication dose.

^*b*^ Gastrointestinal events associated with drug discontinuation or dose changes.

The Hausman test rejected the null hypothesis of equal coefficients in fixed‐effects and random‐effects models (*P* < .001), suggesting a high degree of between‐patient heterogeneity. We therefore report the fixed‐effects model results in Table 3.

**TABLE 3:** Utility decrements for nonfatal events in the safety population^a^ of the ACE trial

	EQ‐5D		EQ‐5D VAS	
	Coefficient (robust SE)	*P* value	Coefficient (robust SE)	*P* value
Short‐term decrements (year of event)				
MI	−0.0210 (0.0106)	.047	−4.0568 (1.0703)	<.001
Stroke	−0.1068 (0.0222)	<.001	−4.5634 (1.7125)	.008
Heart failure	−0.0395 (0.0172)	.022	−3.9796 (1.6718)	.018
Angina	−0.0124 (0.0062)	.047	−1.4871 (0.6437)	.021
Diabetes	−0.0018 (0.0034)	.594	0.2615 (0.3559)	.463
Gastrointestinal^b^	−0,0051 (0.0065)	.430	−0.0916 (0.6231)	.883
Long‐term decrements (event in previous years)				
MI	−0.0308 (0.0112)	.006	−4.2464 (1.2155)	.001
Stroke	−0.0673 (0.0180)	<.001	−2.1093 (1.6293)	.196
Heart failure	−0.0190 (0.0166)	.255	−2.5715 (1.5324)	.940
Time since baseline (year)				
1	0.0017 (0.0017)	.301	−0.1599 (0.1968)	.417
2	0.0027 (0.0019)	.149	−0.4314 (0.1943)	.027
3	0.0032 (0.0021)	.133	−1.0305 (0.2174)	<.001
4	0.0042 (0.0024)	.082	−1.7743 (0.2625)	<.001
5	0.0090 (0.0026)	<.001	−1.9643 (0.3220)	<.001
6	−0.0057 (0.0011)	.108	−2.8453 (0.4110)	<.001
Constant	0.9335 (0.0011)	<.001	83.0840 (0.1184)	<.001
Number of observations	38 114			
Number of patients	6504			
*R* ^2^	0.439		0.478	
*R* ^2^ adjusted	0.323		0.370	

*Note:* Results from fixed‐effects linear regression based on “expected” data (responses recorded during annual visits adjusted for date of annual visit within +/−30 days of “true” [ie, expected] annual visit date) and imputed data for missing responses at annual visits.

Abbreviations: ACE, Acarbose Cardiovascular Evaluation; EQ‐5D, EuroQol‐5 Dimension; MI, myocardial infarction; *R* ^2^, coefficient of determination; SE, standard error; VAS, visual analogue scale.

^a^ Subset of the intention‐to‐treat population who received at least one study medication dose.

^b^ Gastrointestinal events associated with drug discontinuation or dose changes.

The largest decrement in EQ‐5D utility was found for stroke (−0.107; 95% CI, −0.150 to −0.063), followed by heart failure (−0.039; 95% CI, −0.073 to 0.006), MI (0.021; 95% CI, −0.042 to 0.000), and angina (−0.012; 95% CI. −0.025 to 0.000). A history of MI or stroke events was associated with a significant long‐term decrement in EQ‐5D utility in the subsequent years after the event had occurred. We did not find evidence for a statistically significant effect of new‐onset diabetes (−0.002; 95% CI, −0.008 to 0.005) or gastrointestinal events (−0.005; 95% CI, −0.018 to 0.008) on EQ‐5D utility in the year of diagnosis. Time since baseline, independent of complications or baseline age, was positively associated with EQ‐5D utility in the first 5 years of follow‐up. The sensitivity analysis using the EQ‐5D VAS score showed similar results to using the EQ‐5D utility (see Table 3). However, in contrast with the EQ‐5D utility, time since baseline showed a negative association with the VAS score. This is consistent with the observed negative trend in mean EQ‐5D VAS scores (see Figure S3). Table S10, in Supplementary Materials, reports the fixed‐effects models for the EQ‐5D utility and VAS without imputation. The magnitude, direction, and significance of the EQ‐5D utility and VAS decrements were the same as following multiple imputation.

## 4 DISCUSSION

Health utilities in the ACE trial did not differ significantly between the acarbose and placebo arms over time. The majority of trial participants reported “no problems” across health domains throughout the trial. Health‐related quality of life at randomization (mean 0.930, SD 0.104) indicated little or no health problems despite more than 99% of the trial population having a baseline history (per protocol) of cardiovascular events (in terms of MI, stroke, stable/unstable angina, CHF). However, while EQ‐5D utility scores were observed to increase slightly over the first 6 years of the trial period, VAS scores for both treatment arms showed a small decrease within the same period.

The major strength of our analysis is the large, longitudinal data set, with repeated EQ‐5D‐3L measurements for 6522 patients, resulting in around 38 000 person‐year observations. Using a fixed‐effects model, we were able to quantify the impact of major events on health‐related quality of life independent of other patient‐specific effects. We found no evidence for a long‐term impact of heart failure or angina, while results for MI and stroke suggested that patients' quality of life is affected not only in the year of the event but in subsequent years. New‐onset diabetes had no statistically significant impact on health‐related quality of life, possibly reflecting the short follow‐up after diagnosis. Finally, gastrointestinal events associated with drug discontinuation or dose changes had no statistically significant impact on both EQ‐5D utility score and VAS.

The average EQ‐5D utility score for ACE trial participants is similar to results from a recent survey in China among people with established type 2 diabetes (n = 913) that reported a mean EQ‐5D score of 0.986.^30^ The authors of that study suggested that high self‐reported health utilities may be explained by “face‐saving,” whereby Chinese participants tend to provide socially desirable responses. Although the EQ‐5D measurement tool is deemed a suitable method in a Chinese population, other studies have identified a ceiling effect when measuring health‐related quality of life in Chinese populations.^31^, ^32^, ^33^ This ceiling effect may also explain the low variation in EQ‐5D utility scores observed over time and between trial arms, which resulted in our utility decrements associated with cardiovascular events being slightly smaller than those estimated in other studies.^16^ One study using the EQ‐5D VAS score as a predictor of mortality found that people with type 2 diabetes reporting the same VAS score faced higher mortality risks in Asia compared to established market economies (eg, Australia, Canada, France, Germany, UK, etc).^34^ Our sensitivity analysis using the EQ‐5D VAS reported findings generally consistent with the event‐related utility decrements using EQ‐5D utility scores. We also found EQ‐5D utility scores and VAS to be positively correlated (Pearson's coefficient 0.36) as well as significantly associated after adjusting for events and time trends using a fixed‐effects model. Cross‐sectional studies in Chinese populations with established diabetes found slightly lower health‐related quality of life scores that were even lower with longer durations of diabetes.^35^, ^36^ However, EQ‐5D‐3L and VAS scores in absence of complications^36^ were found to be similar to the ones reported here. Further research is needed both to assess the correlation of EQ‐5D utility and VAS scores in different populations and to better understand the reasons for high self‐reporting of health‐related quality of life in Chinese populations.

Our analyses are not without limitations. We used intention‐to‐treat analysis to compare health utilities between the acarbose and placebo trial arms. This implies the underlying assumption that the noncompliance observed in the trial was likely to reflect clinical practice. Furthermore, there was a considerable proportion of EQ‐5D‐3L data missing and we accounted for this using multiple imputation. This assumed data were missing at random, but we found no strong evidence to contradict this assumption. Furthermore, the qualitative conclusions concerning the difference in EQ‐5D utility between treatment arms and the impact of events on utility were the same following multiple imputation and using only available data. Another limitation is the relatively short follow‐up period. Due to little variation in EQ‐5D values over time, our analyses considered first cardiovascular events that were recorded during the trial period and did not account for multiple occurrences of the same event during the first 6 years of the trial follow‐up period or a potential impact of a baseline history. Ideally, the fixed‐effects model would have differentiated between first and second cardiovascular events and controlled for the long‐term history of events when estimating utility decrements. However, due to the relatively small number of events recorded during the trial follow‐up, our analyses lacked the power to adjust the regression model accordingly. Especially for MI and angina, our estimates may underestimate the “true” effect on EQ‐5D utility in the year of the event given that the majority of patients reported having a history of these events at baseline. However, previous studies examining the long‐term impact of MI and angina on health‐related quality of life found it to be negligible.^15^ Our findings suggest the short‐term impact of MI on EQ‐5D utility to be similar to its long‐term decrement in the subsequent years after the event had occurred. Furthermore, our results are based on an “at‐risk” population with a history of CHD at baseline and may therefore not be generalizable to populations without any complications. Finally, most cardiovascular events recorded during the trial follow‐up period occurred before or shortly after the diagnosis of diabetes. Hence, despite the large number of observations available for our analyses, most patients who progressed to diabetes did so in the later years of follow‐up, providing insufficient patient‐year data to estimate any direct effect of diabetes diagnosis on quality of life or any mediating impact of diabetes on utility decrements associated with cardiovascular events.

In conclusion, our results provide estimates of decrements in health‐related quality of life associated with several cardiovascular events in populations with IGT. These will help to inform the development of decision models evaluating the cost‐effectiveness of interventions in populations with IGT. Furthermore, the utility decrements estimated in this study will be used in work assessing the lifetime cost‐effectiveness and quality‐adjusted life years associated with acarbose treatment for people with IGT and CHD.

## CONFLICT OF INTEREST

Rury R. Holman reports grants from Bayer AG during the conduct of the study, personal fees from Amgen, grants from AstraZeneca, personal fees from Bayer, grants and personal fees from Boehringer Ingelheim, other from Elcelyx, other from GSK, other from Janssen, personal fees from Servier, other from Takeda, and grants and personal fees from Merck Sharp & Dohme, outside the submitted work. Jose Leal, Frauke Becker, Lee‐Ling Lim, and Alastair M. Gray report no conflicts of interest.

## AUTHOR CONTRIBUTIONS

All authors contributed to the manuscript and read and approved its final version. Jose Leal designed the study, analyzed the data, drafted the manuscript, and is the guarantor of this work. Frauke Becker analyzed the data and drafted the manuscript. Alastair M. Gray, Lee‐Ling Lim, and Rury R. Holman provided guidance on analyses and drafted the manuscript. Jose Leal and Frauke Becker equally contributed to the work.

## Supporting information


**Appendix S1** Supporting Information
