# Supplementary material for: Health utilities in Chinese patients with coronary heart disease and impaired glucose tolerance (ACE): A longitudinal analysis of a randomized, double‐blind, placebo‐controlled trial
Source: J Diabetes. 2022 Jul 25;14(7):455–64. doi: 10.1111/1753-0407.13294 (PMC9310045; doi:10.1111/1753-0407.13294)
Supplement: Supplementary file 1 — Appendix S1 Supporting Information [file JDB-14-455-s001.docx]

**SUPPLEMENTARY MATERIAL**

We report here additional results of our manuscript. The order of Tables and Figures follows their citation in the manuscript. For convenience, we list below the page number of each Figure and Table.

**List of Figures**

[**Figure S1:** EQ-5D-3L scores (mean, 95% CI) from baseline to year 6 of trial follow-up based on expected data* 9](#_Toc99781702)

[**Figure S2:** EQ-5D-3L scores (mean, 95% CI) from baseline to year 6 of trial follow-up based on expected data and imputed data* 11](#_Toc99781703)

[**Figure S3:** EQ-5D VAS scores (mean, 95% CI) from baseline to year 6 of trial follow-up based on observed data as recorded during for routine visits. 13](#_Toc99781704)

**List of Tables**

[**Table S1:** EQ-5D-3L responses - mobility. 2](#_Toc99782510)

[**Table S2**: EQ-5D-3L responses - self-care. 3](#_Toc99782511)

[**Table S3**: EQ-5D-3L responses – usual activities. 4](#_Toc99782512)

[**Table S4:** EQ-5D-3L responses - pain/discomfort. 5](#_Toc99782513)

[**Table S5:** EQ-5D-3L responses - anxiety/depression. 6](#_Toc99782514)

[**Table S6:** EQ-5D-3L utility scores (observed data). 7](#_Toc99782515)

[**Table S7:** EQ-5D-3L utility scores (expected data). 8](#_Toc99782516)

[**Table S8:** EQ-5D-3L utility scores (expected & imputed data). 10](#_Toc99782517)

[**Table S9:** EQ-5D VAS scores (observed data). 12](#_Toc99782518)

[**Table S10:** Utility decrements for non-fatal events in the safety population of the ACE trial (using available cases) 14](#_Toc99782519)

**Table S1:** EQ-5D-3L responses - mobility.

| **Mobility** |  | Intervention | | Placebo | |
| --- | --- | --- | --- | --- | --- |
|  |  | n | % | n | % |
| Baseline |  | 3,272 |  | 3,250 |  |
|  | No problems | 2,964 | 94 | 2,944 | 94 |
|  | Some problem | 174 | 6 | 187 | 6 |
|  | Severe problem | 5 | 0 | 4 | 0 |
|  | Missing | 129 |  | 115 |  |
| 12 months |  | 3,190 |  | 3,156 |  |
|  | No problems | 2,304 | 94 | 2,218 | 93 |
|  | Some problem | 140 | 6 | 158 | 7 |
|  | Severe problem | 2 | 0 | 2 | 0 |
|  | Missing | 744 |  | 778 |  |
| 24 months |  | 2,923 |  | 2,912 |  |
|  | No problems | 1,927 | 93 | 1,905 | 94 |
|  | Some problem | 131 | 6 | 122 | 6 |
|  | Severe problem | 3 | 0 | 3 | 0 |
|  | Missing | 862 |  | 882 |  |
| 36 months |  | 2,666 |  | 2,644 |  |
|  | No problems | 1,650 | 94 | 1,578 | 93 |
|  | Some problem | 112 | 6 | 116 | 7 |
|  | Severe problem | 0 | 0 | 4 | 0 |
|  | Missing | 904 |  | 946 |  |
| 48 months |  | 2,234 |  | 2,202 |  |
|  | No problems | 1,246 | 92 | 1,259 | 94 |
|  | Some problem | 101 | 7 | 82 | 6 |
|  | Severe problem | 4 | 0 | 0 | 0 |
|  | Missing | 883 |  | 861 |  |
| 60 months |  | 1,623 |  | 1,604 |  |
|  | No problems | 897 | 93 | 883 | 95 |
|  | Some problem | 71 | 7 | 47 | 5 |
|  | Severe problem | 1 | 0 | 0 | 0 |
|  | Missing | 654 |  | 674 |  |
| 72 months |  | 791 |  | 809 |  |
|  | No problems | 452 | 90 | 446 | 91 |
|  | Some problem | 48 | 10 | 41 | 8 |
|  | Severe problem | 1 | 0 | 1 | 0 |
|  | Missing | 290 |  | 321 |  |

n – number of observations.

**Table S2**: EQ-5D-3L responses - self-care.

| **Self care** |  | Intervention | | Placebo | |
| --- | --- | --- | --- | --- | --- |
|  |  | n | % | n | % |
| Baseline |  | 3,272 |  | 3,250 |  |
|  | No problems | 3,072 | 98 | 3,085 | 98 |
|  | Some problem | 64 | 2 | 50 | 2 |
|  | Severe problem | 5 | 0 | 4 | 0 |
|  | Missing | 131 |  | 111 |  |
| 12 months |  | 3,190 |  | 3,156 |  |
|  | No problems | 2,409 | 98 | 2,347 | 99 |
|  | Some problem | 41 | 2 | 33 | 1 |
|  | Severe problem | 2 | 0 | 1 | 0 |
|  | Missing | 738 |  | 775 |  |
| 24 months |  | 2,923 |  | 2,912 |  |
|  | No problems | 2,024 | 98 | 1,990 | 98 |
|  | Some problem | 34 | 2 | 30 | 1 |
|  | Severe problem | 4 | 0 | 3 | 0 |
|  | Missing | 861 |  | 889 |  |
| 36 months |  | 2,666 |  | 2,644 |  |
|  | No problems | 1,738 | 98 | 1,652 | 98 |
|  | Some problem | 28 | 2 | 37 | 2 |
|  | Severe problem | 1 | 0 | 4 | 0 |
|  | Missing | 899 |  | 951 |  |
| 48 months |  | 2,234 |  | 2,202 |  |
|  | No problems | 1,315 | 98 | 1,310 | 98 |
|  | Some problem | 26 | 2 | 31 | 2 |
|  | Severe problem | 3 | 0 | 1 | 0 |
|  | Missing | 890 |  | 860 |  |
| 60 months |  | 1,623 |  | 1,604 |  |
|  | No problems | 950 | 98 | 918 | 99 |
|  | Some problem | 18 | 1 | 12 | 1 |
|  | Severe problem | 1 | 0 | 1 | 0 |
|  | Missing | 654 |  | 673 |  |
| 72 months |  | 791 |  | 809 |  |
|  | No problems | 491 | 98 | 478 | 98 |
|  | Some problem | 9 | 2 | 10 | 2 |
|  | Severe problem | 1 | 0 | 2 | 0 |
|  | Missing | 290 |  | 319 |  |

n – number of observations.

**Table S3**: EQ-5D-3L responses – usual activities.

| **Usual activities** |  | Intervention | | Placebo | |
| --- | --- | --- | --- | --- | --- |
|  |  | n | % | n | % |
| Baseline |  | 3,272 |  | 3,250 |  |
|  | No problems | 2,932 | 93 | 2,928 | 94 |
|  | Some problem | 201 | 6 | 193 | 6 |
|  | Severe problem | 6 | 0 | 8 | 0 |
|  | Missing | 133 |  | 121 |  |
| 12 months |  | 3,190 |  | 3,156 |  |
|  | No problems | 2,329 | 95 | 2,240 | 94 |
|  | Some problem | 117 | 5 | 133 | 6 |
|  | Severe problem | 2 | 0 | 1 | 0 |
|  | Missing | 742 |  | 782 |  |
| 24 months |  | 2,923 |  | 2,912 |  |
|  | No problems | 1,944 | 94 | 1,916 | 95 |
|  | Some problem | 109 | 5 | 105 | 5 |
|  | Severe problem | 6 | 0 | 5 | 0 |
|  | Missing | 864 |  | 886 |  |
| 36 months |  | 2,666 |  | 2,644 |  |
|  | No problems | 1,681 | 95 | 1,589 | 94 |
|  | Some problem | 84 | 5 | 99 | 6 |
|  | Severe problem | 0 | 0 | 6 | 0 |
|  | Missing | 901 |  | 950 |  |
| 48 months |  | 2,234 |  | 2,202 |  |
|  | No problems | 1,270 | 94 | 1,266 | 95 |
|  | Some problem | 74 | 5 | 67 | 5 |
|  | Severe problem | 3 | 0 | 3 | 0 |
|  | Missing | 887 |  | 866 |  |
| 60 months |  | 1,623 |  | 1,604 |  |
|  | No problems | 904 | 94 | 888 | 96 |
|  | Some problem | 62 | 6 | 40 | 4 |
|  | Severe problem | 0 | 0 | 1 | 0 |
|  | Missing | 657 |  | 675 |  |
| 72 months |  | 791 |  | 809 |  |
|  | No problems | 470 | 94 | 461 | 94 |
|  | Some problem | 26 | 5 | 26 | 5 |
|  | Severe problem | 2 | 0 | 3 | 1 |
|  | Missing | 293 |  | 319 |  |

n – number of observations.

**Table S4:** EQ-5D-3L responses - pain/discomfort.

| **Pain/Discomfort** |  | Intervention | | Placebo | |
| --- | --- | --- | --- | --- | --- |
|  |  | n | % | n | % |
| Baseline |  | 3,272 |  | 3,250 |  |
|  | No problems | 2,281 | 73 | 2,259 | 72 |
|  | Some problem | 840 | 27 | 849 | 27 |
|  | Severe problem | 19 | 1 | 9 | 0 |
|  | Missing | 132 |  | 133 |  |
| 12 months |  | 3,190 |  | 3,156 |  |
|  | No problems | 1,786 | 74 | 1,702 | 72 |
|  | Some problem | 631 | 26 | 653 | 28 |
|  | Severe problem | 11 | 0 | 9 | 0 |
|  | Missing | 762 |  | 792 |  |
| 24 months |  | 2,923 |  | 2,912 |  |
|  | No problems | 1,498 | 73 | 1,446 | 72 |
|  | Some problem | 533 | 26 | 556 | 28 |
|  | Severe problem | 12 | 1 | 10 | 0 |
|  | Missing | 880 |  | 900 |  |
| 36 months |  | 2,666 |  | 2,644 |  |
|  | No problems | 1,277 | 73 | 1,237 | 73 |
|  | Some problem | 477 | 27 | 445 | 26 |
|  | Severe problem | 7 | 0 | 7 | 0 |
|  | Missing | 905 |  | 955 |  |
| 48 months |  | 2,234 |  | 2,202 |  |
|  | No problems | 988 | 74 | 967 | 72 |
|  | Some problem | 348 | 26 | 368 | 28 |
|  | Severe problem | 7 | 1 | 2 | 0 |
|  | Missing | 891 |  | 865 |  |
| 60 months |  | 1,623 |  | 1,604 |  |
|  | No problems | 729 | 76 | 680 | 73 |
|  | Some problem | 228 | 24 | 243 | 26 |
|  | Severe problem | 8 | 1 | 4 | 0 |
|  | Missing | 658 |  | 677 |  |
| 72 months |  | 791 |  | 809 |  |
|  | No problems | 371 | 75 | 348 | 72 |
|  | Some problem | 126 | 25 | 134 | 28 |
|  | Severe problem | 0 | 0 | 3 | 1 |
|  | Missing | 294 |  | 324 |  |

n – number of observation.

**Table S5:** EQ-5D-3L responses - anxiety/depression.

| **Anxiety/Depression** |  | Intervention | | Placebo | |
| --- | --- | --- | --- | --- | --- |
|  |  | n | % | n | % |
| Baseline |  | 3,272 |  | 3,250 |  |
|  | No problems | 2644 | 84 | 2626 | 84 |
|  | Some problem | 490 | 16 | 494 | 16 |
|  | Severe problem | 7 | 0 | 8 | 0 |
|  | Missing | 131 |  | 122 |  |
| 12 months |  | 3,190 |  | 3,156 |  |
|  | No problems | 2095 | 86 | 2015 | 85 |
|  | Some problem | 341 | 14 | 345 | 15 |
|  | Severe problem | 6 | 0 | 5 | 0 |
|  | Missing | 748 |  | 791 |  |
| 24 months |  | 2,923 |  | 2,912 |  |
|  | No problems | 1776 | 86 | 1743 | 86 |
|  | Some problem | 272 | 13 | 271 | 13 |
|  | Severe problem | 10 | 0 | 10 | 0 |
|  | Missing | 865 |  | 888 |  |
| 36 months |  | 2,666 |  | 2,644 |  |
|  | No problems | 1543 | 88 | 1471 | 87 |
|  | Some problem | 214 | 12 | 209 | 12 |
|  | Severe problem | 5 | 0 | 9 | 1 |
|  | Missing | 904 |  | 955 |  |
| 48 months |  | 2,234 |  | 2,202 |  |
|  | No problems | 1183 | 88 | 1178 | 88 |
|  | Some problem | 153 | 11 | 157 | 12 |
|  | Severe problem | 8 | 1 | 1 | 0 |
|  | Missing | 890 |  | 866 |  |
| 60 months |  | 1,623 |  | 1,604 |  |
|  | No problems | 863 | 89 | 836 | 90 |
|  | Some problem | 100 | 10 | 85 | 9 |
|  | Severe problem | 3 | 0 | 4 | 0 |
|  | Missing | 657 |  | 679 |  |
| 72 months |  | 791 |  | 809 |  |
|  | No problems | 455 | 92 | 437 | 90 |
|  | Some problem | 39 | 8 | 49 | 10 |
|  | Severe problem | 2 | 0 | 1 | 0 |
|  | Missing | 295 |  | 322 |  |

n – number of observations.

**Table S6:** EQ-5D-3L utility scores (observed data*).

| **EQ-5D-3L** | | Intervention | | Placebo | | All subjects | |
| --- | --- | --- | --- | --- | --- | --- | --- |
|  |  | n=3,272 | | n=3,250 | | n=6,522 | |
| *Baseline* | |  |  |  |  |  |  |
|  | n | 3,272 |  | 3,250 |  | 6,522 |  |
|  | n (complete data) | 3,080 |  | 3,067 |  | 6,147 |  |
|  | Mean (SD) | 0.933 | (0.107) | 0.934 | (0.107) | 0.933 | (0.107) |
|  | n (missing data) (%) | 192 | (5.9) | 183 | (5.6) | 375 | (5.7) |
| *Year 1* | |  |  |  |  |  |  |
|  | n | 3,190 |  | 3,156 |  | 6,346 |  |
|  | n (complete data) | 2,400 |  | 2,329 |  | 4,729 |  |
|  | Mean (SD) | 0.938 | (0.100) | 0.934 | (0.104) | 0.936 | (0.102) |
|  | n (missing data) (%) | 790 | (24.8) | 827 | (26.2) | 1,617 | (25.5) |
| *Year 2* | |  |  |  |  |  |  |
|  | n | 2,923 |  | 2,912 |  | 5,835 |  |
|  | n (complete data) | 2,020 |  | 1,989 |  | 4,009 |  |
|  | Mean (SD) | 0.936 | (0.110) | 0.935 | (0.106) | 0.936 | (0.108) |
|  | n (missing data) (%) | 903 | (30.9) | 923 | (31.7) | 1,826 | (31.3) |
| *Year 3* | |  |  |  |  |  |  |
|  | n | 2,666 |  | 2,644 |  | 5,310 |  |
|  | n (complete data) | 1,744 |  | 1,667 |  | 3,411 |  |
|  | Mean (SD) | 0.938 | (0.099) | 0.935 | (0.113) | 0.937 | (0.106) |
|  | n (missing data) (%) | 922 | (34.6) | 977 | (37.0) | 1,899 | (35.8) |
| *Year 4* | |  |  |  |  |  |  |
|  | n | 2,234 |  | 2,202 |  | 4,436 |  |
|  | n (complete data) | 1,322 |  | 1,317 |  | 2,639 |  |
|  | Mean (SD) | 0.936 | (0.111) | 0.939 | (0.101) | 0.937 | (0.106) |
|  | n (missing data) (%) | 912 | (40.8) | 885 | (40.2) | 1,797 | (40.5) |
| *Year 5* | |  |  |  |  |  |  |
|  | n | 1,623 |  | 1,604 |  | 3,227 |  |
|  | n (complete data) | 953 |  | 918 |  | 1,871 |  |
|  | Mean (SD) | 0.941 | (0.103) | 0.944 | (0.095) | 0.942 | (0.099) |
|  | n (missing data) (%) | 670 | (41.3) | 686 | (42.8) | 1,356 | (42.0) |
| *Year 6* | |  |  |  |  |  |  |
|  | n | 791 |  | 809 |  | 1,600 |  |
|  | n (complete data) | 490 |  | 480 |  | 970 |  |
|  | Mean (SD) | 0.941 | (0.105) | 0.935 | (0.116) | 0.938 | (0.111) |
|  | n (missing data) (%) | 301 | (38.1) | 329 | (40.7) | 630 | (39.4) |

* Responses recorded during annual visits. n – number of observations; SD – standard deviation.

**Table S7:** EQ-5D-3L utility scores (expected data*).

| **EQ-5D-3L** | | | Intervention | | | | Placebo | | | | All subjects | | | |
| --- | --- | --- | --- | --- | --- | --- | --- | --- | --- | --- | --- | --- | --- | --- |
|  |  | | n=3,272 | | | | n=3,250 | | | | n=6,522 | | | |
| *Baseline* | | |  | |  | |  | |  | |  | |  | |
|  | n | | 3,272 | |  | | 3,250 | |  | | 6,522 | |  | |
|  | n (data available) | | 3,272 | |  | | 3,250 | |  | | 6,522 | |  | |
|  | Mean (SD) | | 0.933 | | (0.104) | | 0.934 | | (0.104) | | 0.933 | | (0.104) | |
|  | n (missing data) (%) | | 0 | | (0.0) | | 0 | | (0.0) | | 0 | | (0.0) | |
| *Year 1* | | |  | |  | |  | |  | |  | |  | |
|  | n | | 3,190 | |  | | 3,156 | |  | | 6,346 | |  | |
|  | n (data available) | | 2,410 | |  | | 2,337 | |  | | 4,747 | |  | |
|  | Mean (SD) | | 0.938 | | (0.100) | | 0.934 | | (0.104) | | 0.936 | | (0.102) | |
|  | n (missing data) (%) | | 780 | | (24.5) | | 819 | | (26.0) | | 1,599 | | (25.2) | |
| *Year 2* | | |  | |  | |  | |  | |  | |  | |
|  | n | | 2,923 | |  | | 2,912 | |  | | 5,835 | |  | |
|  | n (data available) | | 1,970 | |  | | 1,943 | |  | | 3,913 | |  | |
|  | Mean (SD) | | 0.937 | | (0.108) | | 0.935 | | (0.105) | | 0.936 | | (0.107) | |
|  | n (missing data) (%) | | 953 | | (32.6) | | 969 | | (33.3) | | 1,922 | | (32.9) | |
| *Year 3* | | |  | |  | |  | |  | |  | |  | |
|  | n | | 2,666 | |  | | 2,644 | |  | | 5,310 | |  | |
|  | n (data available) | | 1,725 | |  | | 1,630 | |  | | 3,355 | |  | |
|  | Mean (SD) | | 0.938 | | (0.099) | | 0.936 | | (0.112) | | 0.937 | | (0.105) | |
|  | n (missing data) (%) | | 941 | | (35.3) | | 1,014 | | (38.4) | | 1,955 | | (36.8) | |
| *Year 4* | | |  | |  | |  | |  | |  | |  | |
|  | n | | 2,234 | |  | | 2,202 | |  | | 4,436 | |  | |
|  | n (data available) | | 1,321 | |  | | 1,297 | |  | | 2,618 | |  | |
|  | Mean (SD) | | 0.938 | | (0.107) | | 0.940 | | (0.097) | | 0.939 | | (0.102) | |
|  | n (missing data) (%) | | 913 | | (40.9) | | 905 | | (41.1) | | 1,818 | | (41.0) | |
| *Year 5* | | |  | |  | |  | |  | |  | |  | |
|  | n | | 1,623 | |  | | 1,604 | |  | | 3,227 | |  | |
|  | n (data available) | | 926 | |  | | 896 | |  | | 1,822 | |  | |
|  | Mean (SD) | | 0.942 | | (0.099) | | 0.944 | | (0.094) | | 0.943 | | (0.097) | |
|  | n (missing data) (%) | | 697 | | (42.9) | | 708 | | (44.1) | | 1,405 | | (43.5) | |
| *Year 6* | | |  | |  | |  | |  | |  | |  | |
|  | n | | 791 | |  | | 809 | |  | | 1,600 | |  | |
|  | n (data available) | | 440 | |  | | 458 | |  | | 898 | |  | |
|  | Mean (SD) | | 0.943 | | (0.104) | | 0.936 | | (0.116) | | 0.939 | | (0.110) | |
|  | | n (missing data) (%) | | 351 | | (44.4) | | 351 | | (43.4) | | 70 | | (43.9) |

* Expected data from responses recorded during annual visits adjusted for date of annual visit within +/- 30 days of ‘true’ annual visit date. n – number of observations; SD – standard deviation.

**Figure S1:** EQ-5D-3L scores (mean, 95% CI) from baseline to year 6 of trial follow-up based on expected data*

*responses recorded during annual visits, adjusted for date of annual visit within +/- 30 days of ‘true’ annual visit date, observed data as recorded during routine visits; no imputation of missing data

**Table S8:** EQ-5D-3L utility scores (expected & imputed data*).

| **EQ-5D-3L** | | Intervention | | Placebo | | All subjects | |
| --- | --- | --- | --- | --- | --- | --- | --- |
|  |  | n=3,272 | | n=3,250 | | n=6,522 | |
| *Baseline* | |  |  |  |  |  |  |
|  | n | 3,272 |  | 3,250 |  | 6,522 |  |
|  | Mean (SE) | 0.933 | (0.002) | 0.934 | (0.002) | 0.933 | (0.001) |
|  |  |  |  |  |  |  |  |
| *Year 1* | |  |  |  |  |  |  |
|  | n | 3,190 |  | 3,156 |  | 6,346 |  |
|  | Mean (SE) | 0.936 | (0.002) | 0.933 | (0.002) | 0.935 | (0.001) |
|  |  |  |  |  |  |  |  |
| *Year 2* | |  |  |  |  |  |  |
|  | N | 2,923 |  | 2,912 |  | 5,835 |  |
|  | Mean (SE) | 0.935 | (0.002) | 0.934 | (0.002) | 0.935 | (0.002) |
|  |  |  |  |  |  |  |  |
| *Year 3* | |  |  |  |  |  |  |
|  | N | 2,666 |  | 2,644 |  | 5,310 |  |
|  | Mean (SE) | 0.936 | (0.002) | 0.933 | (0.003) | 0.934 | (0.002) |
|  |  |  |  |  |  |  |  |
| *Year 4* | |  |  |  |  |  |  |
|  | N | 2,234 |  | 2,202 |  | 4,436 |  |
|  | Mean (SE) | 0.936 | (0.003) | 0.937 | (0.003) | 0.937 | (0.002) |
|  |  |  |  |  |  |  |  |
| *Year 5* | |  |  |  |  |  |  |
|  | N | 1,623 |  | 1,604 |  | 3,227 |  |
|  | Mean (SE) | 0.940 | (0.004) | 0.939 | (0.003) | 0.939 | (0.003) |
|  |  |  |  |  |  |  |  |
| *Year 6* | |  |  |  |  |  |  |
|  | N | 791 |  | 809 |  | 1,600 |  |
|  | Mean (SE) | 0.934 | (0.006) | 0.928 | (0.088) | 0.931 | (0.004) |

* Expected data (responses recorded during annual visits adjusted for date of annual visit within +/- 30 days of ‘true’ annual visit date) and imputed data for missing responses at annual visits. SD – standard deviation.

**Figure S2:** EQ-5D-3L scores (mean, 95% CI) from baseline to year 6 of trial follow-up based on expected data and imputed data*

***** responses recorded during annual visits adjusted for date of annual visit within +/- 30 days of ‘true’ annual visit date, observed data as recorded during for routine visits) and imputed data for annual visits.

**Table S9:** EQ-5D VAS scores (observed data*).

| **EQ-5D VAS** | | Intervention | | Placebo | | All subjects | |
| --- | --- | --- | --- | --- | --- | --- | --- |
|  |  | n=3,272 | | n=3,250 | | n=6,522 | |
| *Baseline* | |  |  |  |  |  |  |
|  | n | 3,272 |  | 3,250 |  | 6,522 |  |
|  | n (complete data) | 3,092 |  | 3,078 |  | 6,170 |  |
|  | Mean (SD) | 83.0 | (12.2) | 82.9 | (11.9) | 82.9 | (12.0) |
|  | n (missing data) (%) | 180 | (5.5) | 172 | (5.3) | 352 | (5.4) |
| *Year 1* | |  |  |  |  |  |  |
|  | n | 3,190 |  | 3,146 |  | 6,346 |  |
|  | n (complete data) | 2,413 |  | 2,343 |  | 4,756 |  |
|  | Mean (SD) | 83.0 | (11.6) | 82.5 | (11.8) | 82.8 | (11.7) |
|  | n (missing data) (%) | 777 | (24.4) | 813 | (25.8) | 1,590 | (25.1) |
| *Year 2* | |  |  |  |  |  |  |
|  | n | 2,923 |  | 2,912 |  | 5,835 |  |
|  | n (complete data) | 2,027 |  | 1,986 |  | 4,013 |  |
|  | Mean (SD) | 82.7 | (11.2) | 82.4 | (11.0) | 82.6 | (11.1) |
|  | n (missing data) (%) | 896 | (30.7) | 926 | (31.8) | 1,822 | (31.2) |
| *Year 3* | |  |  |  |  |  |  |
|  | n | 2,666 |  | 2,644 |  | 5,310 |  |
|  | n (complete data) | 1,725 |  | 1,674 |  | 3,399 |  |
|  | Mean (SD) | 82.1 | (11.2) | 82.0 | (11.2) | 82.1 | (11.2) |
|  | n (missing data) (%) | 941 | (35.3) | 970 | (36.7) | 1,911 | (36.0) |
| *Year 4* | |  |  |  |  |  |  |
|  | n | 2,234 |  | 2,202 |  | 4,436 |  |
|  | n (complete data) | 1,330 |  | 1,330 |  | 2,660 |  |
|  | Mean (SD) | 81.4 | (11.5) | 81.4 | (11.3) | 81.4 | (11.4) |
|  | n (missing data) (%) | 904 | (40.5) | 872 | (39.6) | 1,776 | (40.0) |
| *Year 5* | |  |  |  |  |  |  |
|  | n | 1,623 |  | 1,604 |  | 3,227 |  |
|  | n (complete data) | 958 |  | 922 |  | 1,880 |  |
|  | Mean (SD) | 81.8 | (11.7) | 81.9 | (11.3) | 81.8 | (11.5) |
|  | n (missing data) (%) | 665 | (41.0) | 682 | (42.5) | 1,347 | (41.7) |
| *Year 6* | |  |  |  |  |  |  |
|  | n | 791 |  | 808 |  | 1,600 |  |
|  | n (complete data) | 500 |  | 484 |  | 984 |  |
|  | Mean (SD) | 81.6 | (11.5) | 81.6 | (11.5) | 81.6 | (11.7) |
|  | n (missing data) (%) | 291 | (36.8) | 325 | (40.2) | 616 | (38.5) |

* Responses recorded during annual visits. VAS – visual analogue scale. SD – standard deviation.

**Figure S3:** EQ-5D VAS scores (mean, 95% CI) from baseline to year 6 of trial follow-up based on observed data as recorded during for routine visits.

**Table S10:** Utility decrements for non-fatal events in the safety population* of the ACE trial (using available cases)

|  | **EQ-5D utility** | | **EQ-5D VAS** | |
| --- | --- | --- | --- | --- |
|  | Coefficient (robust SE) | p-value | Coefficient (robust SE) | p-value |
| Short-term decrements (year of event) | |  |  |  |
| MI | -0.0228 (0.0102) | 0.0260 | -4.7988 (1.1372) | <0.0001 |
| Stroke | -0.1037 (0.0302) | 0.0010 | -3.8371 (1.4822) | 0.0100 |
| Heart failure | -0.0447 (0.0210) | 0.0330 | -4.9503 (1.7414) | 0.0040 |
| Angina | -0.0133 (0.0062) | 0.0330 | -1.5403 (0.6837) | 0.0240 |
| Diabetes | -0.0023 (0.0033) | 0.4760 | -0.0261 (0.3259) | 0.9360 |
| Gastrointestinal** | -0.0017 (0.0062) | 0.7840 | -0.0769 (0.5997) | 0.8980 |
| Long-term decrements (event in previous years) | |  |  |  |
| MI | -0.0300 (0.0118) | 0.0110 | -4.6999 (1.1560) |  |
| Stroke | -0.0801 (0.0205) | <0.0001 | -0.7328 (1.5671) | 0.6400 |
| Heart failure | -0.0218 (0.0154) | 0.1560 | -4.0412 (1.6083) | 0.1200 |
| Time since baseline (year) | | | | |
| 1 | 0.0017 (0.0016) | 0.2850 | -0.2252 (0.1840) | 0.2210 |
| 2 | 0.0026 (0.0019) | 0.1690 | -0.4959 (0.1938) | 0.0110 |
| 3 | 0.0035 (0.0020) | 0.0750 | -1.1365 (0.2034) | <0.0001 |
| 4 | 0.0042 (0.0021) | 0.0490 | -1.7561 (0.2324) | <0.0001 |
| 5 | 0.0085 (0.0024) | <0.0001 | -1.7715 (0.2666) | <0.0001 |
| 6 | 0.0025 (0.0034) | 0.4650 | -2.4434 (0.3263) | <0.0001 |
| Constant | 0.9352 (0.0010) | <0.0001 | 83.2893 (0.1151) | <0.0001 |
| Number of observations | 23799 |  | 23781 |  |
| Number of patients | 6504 |  | 6504 |  |

Results from fixed-effects linear regression based on ‘expected’ data (responses recorded during annual visits adjusted for date of annual visit within +/- 30 days of ‘true’ annual visit date) and observed data as recorded during routine visits. MI – myocardial infarction. SE – standard error. *Subset of the intention-to-treat population who received at least one study medication dose*.* **Gastrointestinal events associated with drug discontinuation or dose changes*.*
